# Applications of FAPI PET/CT in the diagnosis and treatment of breast and the most common gynecologic malignancies: a literature review

**DOI:** 10.3389/fonc.2024.1358070

**Published:** 2024-03-05

**Authors:** Tengfei Li, Jintao Zhang, Yuanzhuo Yan, Min Tan, Yue Chen

**Affiliations:** ^1^ Department of Nuclear Medicine, The Affiliated Hospital of Southwest Medical University, Luzhou, Sichuan, China; ^2^ Nuclear Medicine and Molecular Imaging Key Laboratory of Sichuan Province, Luzhou, Sichuan, China; ^3^ Nuclear Medicine Institute of Southwest Medical University, Luzhou, Sichuan, China

**Keywords:** FAPI, breast cancer, gynecologic malignancies, ovarian cancer, cervical cancer, endometrial cancer, PET/CT

## Abstract

The fibroblast activating protein (FAP) is expressed by some fibroblasts found in healthy tissues. However, FAP is overexpressed in more than 90% of epithelial tumors, including breast and gynecological tumors. As a result, the FAP ligand could be used as a target for diagnosis and treatment purposes. Positron emission tomography/computed tomography (PET/CT) is a hybrid imaging technique commonly used to locate and assess the tumor’s molecular and metabolic functions. PET imaging involves the injection of a radiotracer that tends to accumulate more in metabolically active lesions such as cancer. Several radiotracers have been developed to target FAP in PET/CT imaging, such as the fibroblast-activation protein inhibitor (FAPI). These tracers bind to FAP with high specificity and affinity, allowing for the non-invasive detection and quantification of FAP expression in tumors. In this review, we discussed the applications of FAPI PET/CT in the diagnosis and treatment of breast and the most common gynecologic malignancies. Radiolabeled FAPI can improve the detection, staging, and assessment of treatment response in breast and the most common gynecologic malignancies, but the problem with normal hormone-responsive organs remains insurmountable. Compared to the diagnostic applications of FAPI, further research is needed for future therapeutic applications.

## Introduction

Positron emission tomography/computed tomography (PET/CT) is an imaging technique used to obtain anatomical, functional, and molecular information about the tumor. It plays an important role in the diagnosis of malignant tumors and is widely used to detect and stage cancer (1). 18-Fluorodeoxyglucose (18F-FDG) is the most commonly used radiotracer for PET/CT imaging. 18F-FDG has a high sensitivity and specificity to detect, stage, and assess treatment response in cancer patients. However, ^18^F-FDG also has a number of limitations. This radiotracer has a relatively low sensitivity for the detection of micrometastases and lymph nodes less than 1cm and it can not accurately distinguish between acute inflammatory infections from tumor growth (2, 3). Moreover, patients need to fast before the examination, and the whole examination can take up to a longer scanning time. As a result, new radiotracers are being developed to improve the diagnostic accuracy and facilitate the examination process of PET/CT.

The fibroblast activation protein (FAP) is a membrane-bound serine protease that is expressed by activated fibroblasts and other cell types in the tumor microenvironment. FAP has both gelatinase and dipeptidyl peptidase activities, allowing it to cleave a variety of substrates, including type I collagen and dipeptides. Therefore, FAP plays a key role in the remodeling of the extracellular matrix and in the formation of fibrous tissues, which are critical components of wound healing and tissue repair processes (4–6). FAP has also been found to be overexpressed in cancer-associated fibroblasts (CAF) found in the tumor microenvironment of various malignant epithelial tumors such as breast, colon, and pancreatic cancer. However, FAP is generally not expressed or expressed at very low levels in normal tissue and benign tumors. Activated CAFs can secrete various factors such as cytokines, growth factors, extracellular matrix proteins, and enzymes. These factors can promote angiogenesis, immune evasion, tumor growth, and progression. As a result, elevated FAP levels are usually linked with a poor prognosis (7).

The fibroblast activation protein inhibitor (FAPI) is a small molecule that can bind with the FAP enzyme domain found on the surface of the CAFs. As a result, FAPI is increasingly being used to diagnose and target cancers with high FAP expression (8). FAPI can be coupled with the chelating agent 1,4,7,10-tetraazacyclododecane-1,4,7,10-tetraacetic acid (DOTA) and the radiotracer gallium-68 (^68^Ga) in PET/CT imaging. FAPI PET/CT imaging shows promising results in cancer imaging, particularly for the most common gynecologic malignancies (9, 10). The FAPI PET/CT examination can be performed in 10 minutes to 1 hour without the need for fasting and resting time. As a result, when compared with ^18^F-FDG PET/CT, FAPI PET/CT has a higher patient throughput (5). ^68^Ga-FAPI is also being used as a radiotracer for PET/CT imaging. ^68^Ga-FAPI has low uptake in normal tissue and high uptake in many cancers, including cancers with low ^18^F-FDG affinity. Compared with ^18^F-FDG PET/CT, ^68^Ga-FAPI has a higher tumor-to-background ratio (TBR) and can therefore facilitate the distinction between normal and cancerous tissue (11–13). On the other hand, FAPI can also be combined with beta-emitting isotopes such as lutetium-177 (^177^Lu) or yttrium-90 (^90^Y) and used to treat malignant tumors with high fibroblastic activity, such as breast cancer and pancreatic cancer (14).

## Biological dose distribution of FAPI

The normal physiological distribution of FAPI in the human body is similar to that of ^18^F-FDG. However, the uptake of radiolabeled FAPI is lower in areas with high physiological ^18^F-FDG uptake, such as the brain. Giesel et al. (5) compared the physiological uptake of two radiolabeled FAPI, ^68^Ga-FAPI-02 and ^68^Ga-FAPI-04, to acquire the PET/CT images in two cancer patients. PET/CT imaging was performed at 0.2 hours, 1 hour, and 3 hours after the radiopharmaceutical injection. The biological distribution of the radiopharmaceutical in the patients was quantified by measuring the mean standard uptake value (SUVmean) and maximum standard uptake value (SUVmax). These FAPI ligands had a lower SUVmax than ^18^F-FDG in the brain (0.32 versus 11.01), liver (1.69 versus 2.77), and oropharyngeal mucosa (2.57 versus 4.88). The TBR of the two radiopharmaceuticals was similar at 1 hour after injection. Meyer et al. (15) showed that ^68^Ga-FAPI-46 PET/CT imaging had a good dose distribution, favorable tracer kinetics, and high diagnostic efficacy in six cancer patients. Other studies have also shown that since the kidney rapidly clears the various radiolabeled FAPI ligands, its uptake in normal organs remains low and only changes slightly over time, thus facilitating the imaging procedure (16, 17). Normal organs and inflamed tissue will have some physiological uptake of FAPI. Some healthy patients will also have high physiological uptake in the breast 60 minutes after the FAPI injection, with an average SUV of 4.5 ± 1.5 (18). In healthy adult tissue FAP is scarcely present, with some exceptions such as during embryogenesis, human placenta and in uterine stroma, particularly during the proliferative phase (19, 20). In addition, some case reports have shown that hormones can stimulate FAP expression in hormone-sensitive organs such as the breast or uterus (21). Therefore, we aimed to explore the application of using radiolabeled FAPI for diagnosing and treating breast and the most common gynecologic malignancies.

## Breast cancer

### Applications of FAPI PET/CT in the diagnosis of breast cancer

Breast cancer has surpassed lung cancer as the most commonly diagnosed cancer, with an estimated 2.3 million new cases, making it the most prominent cancer and the leading cause of cancer-related death among women worldwide (22). Breast cancer often has bone metastasis, which can lead to pain and severe deterioration in the patient’s quality of life (23). Breast cancer can be divided into 4 molecular subtypes, including luminal-A, luminal-B, human epidermal growth factor-2 overexpressed (HER2-OE), and basal-like (Triple-negative breast cancer; TNBC) based on the expression of the Kiel-67 (Ki-67) protein, and the estrogen (ER), progesterone (PR), and HER-2 receptors (24). Luminal A-like cancers highly express the ER and PR receptors (>20%), have low expression levels of Ki-67 (<20%), and do not express the HER-2 receptor. Luminal B-like cancers also express the ER receptor, but they have low expression levels of the PR receptor (<20%). These tumors may also express the HER-2 receptor and have high expression levels of Ki-67 (>20%). The HER2-OE tumors are characterized by overexpressed HER-2 and negative hormone receptors and the basal-like cancers do not express any receptors.

The uptake of 18F-FDG varies between the different molecular subtypes and could be used to facilitate differential diagnosis (25). Groheux D et al. (26) confirmed it, showing significantly higher 18F-FDG uptake in ER− tumours than in ER+ tumours and a significantly higher 18F-FDG uptake in PR− tumours than in PR+ tumours. Besides, triple-negative breast cancer is usually highly 18F-FDG–avid. Although 18F-FDG PET/CT has great value in diagnosing and staging breast cancer, its high physiological background activity in multiple organs and inflamed tissued limits its specificity and leads to a high false positive rate (27).

On the other hand, the background uptake of the radiolabeled FAPI in the brain, liver, and oral mucosa is significantly lower than that of ^18^F-FDG, thus providing a more accurate staging tool for breast cancer staging (17). ^68^Ga-FAPI showed an overall high tumor uptake in breast cancer and had a higher metastatic detection rate than ^18^F-FDG, especially for bone and peritoneal cancer metastasis (28, 29). Ding et al. (13) found in a mouse model (n=40) that compared with ^18^F-FDG, ^68^Ga-FAPI-04 was more sensitive in detecting multiple distant metastases in early-stage breast cancer, but less sensitive than ^18^F-FDG in advanced stage disease. Elboga et al. (30) compared the diagnostic accuracy of ^68^Ga-FAPI-04 and ^18^F-FDG PET/CT in detecting breast cancer based on the SUVmax, and TBRs measurement in 48 breast cancer patients and found that ^68^Ga-FAPI-04 performed better than ^18^F-FDG. Similarly, Komek et al. (31) compared the diagnostic accuracy of ^68^Ga-FAPI-04 with ^18^F-FDG using pathological results as a gold standard in 20 women diagnosed with breast cancer and found that ^68^Ga-FAPI-04 had a superior detection rate for primary breast cancer and metastasis in the lymph node, liver, bone, and brain. However, a retrospective dual-center analysis conducted by Dendl K et al. (11) showed that the SUVmax of the radiolabeled FAPI in the breast of premenopausal women was significantly higher than that of postmenopausal patients. It has certain guiding significance for the differential diagnosis of breast cancer and physiologic uptake in young women. ^68^Ga-FAPI PET/CT still has high false positive and false negative rates caused by the non-tumor-specific uptake in scars, normal breast tissue, inflammatory tissue, and postoperative wound healing (18). Besides, ^68^Ga-FAPI could also improve the detection of metastasis in latent breast cancer due to its favorable physical properties (32, 33).

Although most studies have shown that 68Ga-FAPI PET/CT has an overall advantage in the diagnosis of breast cancer, it has a number of limitations. Moreover, the 68Ga has a short half-life and thus its production requires an onsite cyclotron. This problem can be overcome by using fluorine-18, aluminum fluoride, and 1,4,7-triazacyclononane-1,4,7-triacetic acid (NOTA) to label the FAPI (18F-ALF-NOTA-FAPI-04). Compared with 68Ga, the longer half-life of 18F allows for more flexible imaging protocols and better time management for clinical facilities. The 18F-labeled FAPI also exhibits better *in vivo* stability and pharmacokinetics, which can improve imaging quality and accuracy. In addition, the production and transportation of 18F-labeled tracers are generally more convenient than those of 68Ga, since it does not require an onsite cyclotron for production (34–36).

### Applications of FAPI-PET/CT in the treatment of breast cancer

There are various treatment options for breast cancer, where surgery remains the main option. However, this treatment is often combined with adjuvant and neoadjuvant systemic therapies, including chemotherapy, HER2-directed therapy, and endocrine therapy (37, 38). In recent years, radionuclide therapy has shown promising results in managing breast cancer. Lindner et al. (39) marked FAPI-04 with ^90^Y and used it to treat two patients with metastatic breast cancer. Both patients experienced a significant reduction in pain after administering low doses of the drug. The authors anticipated that higher activities can be given resulting in better tumoricidal effects. These findings indicated that this treatment could effectively manage malignant tumors with highly active fibroblasts, such as breast cancer. In addition, other new FAPI reagents ^177^Lu-DOTA.SA.FAPi and ^177^Lu-DOTAGA.(SA.FAPi)_2_ also show promising results in the treatment of advanced refractory breast cancer (40–42). The histopathology of a breast cancer patient revealed atypical cells arranged cords and tubules in the fibrotic stroma suggestive of invasive ductal carcinoma of the breast and the immunohistochemistry reported cells immunopositive for Her2neu, while negative for ER and PR. As the patient had depleted all approved therapy options, she was counselled for ^177^Lu-DOTA.SA.FAPi therapy. Post-treatment, the patient experienced a decrease in the intensity of headaches. Post-treatment 4-week laboratory parameters were well within the normal range and no treatment-related adverse events were observed. The result showed that ^177^Lu-DOTA.SA.FAPi therapy may open up a new opportunity in breast cancer therapy, particularly for the patients refractory to conventional treatment options (40). Novel radiopharmaceuticals such as ^90^Y-FAPI-46, ^177^Lu-FAP-2286, and ^177^Lu-FAPI-46 are currently being explored as potential new treatments for breast cancer (43–46).

## Ovarian cancer

### Applications of FAPI PET/CT in the diagnosis of ovarian cancer

Ovarian cancer is the fifth most common cause of cancer death in women, behind only to lung, breast, colorectal, and pancreatic cancer. Its incidence and mortality increase with age. Most patients with ovarian cancer are older than 50, but patients can be diagnosed with ovarian cancer at any age (22, 47, 48). The early diagnosis of ovarian cancer is beneficial to the detection of metastases and preoperative staging. The metastasis of ovarian cancer usually includes peritoneal, lymphatic or hematogenous dissemination, which is widely spread through ascites circulation. ^18^F-FDG PET/CT is superior to conventional imaging in detecting small metastases of ovarian cancer, but it also has some limitations. Despite its limitations, ^18^F-FDG remains the most commonly used radiotracer for diagnosing ovarian cancer. However, the accumulation of this radiotracer in normal ovarian tissue often leads to a false positive diagnosis, particularly in premenopausal women (18, 49, 50).

FAP is often highly expressed in the stroma of ovarian cancers and has an important role in the proliferation, invasion, and migration of ovarian cancer cells (51, 52). Therefore, the radiolabeled FAPI could be used to facilitate the diagnosis of ovarian cancer. As opposed to ^18^F-FDG, ^68^Ga-DOTA-FAPI-04 has no uptake in benign ovarian lesions, and its uptake is not affected by the menstrual cycle (53–55). As a result, studies have shown that the ^68^Ga-DOTA-FAPI-04 can accurately distinguish between benign and early-stage malignant ovarian lesions (53). A case report by Siripongsatian D et al. (56) also showed a negative ^18^F-FDG uptake and strong ^68^Ga-FAPI uptake in a recurrent ovarian clear cell carcinoma. In addition, a retrospective study by Zheng et al. (57) in 27 patients with pathologically confirmed ovarian cancer showed that compared with ^18^F-FDG, ^68^Ga-FAPI PET/CT could more accurately differentiate between the different types of malignant ovarian tumors, including high-grade serous carcinomas (HGSC), low-grade serous carcinomas (LGSC), and ovarian mucinous carcinoma. Moreover, Zheng et al. (57) found that ^68^Ga-FAPI PET/CT was more sensitive than ^18^F-FDG PET/CT in the detection of primary ovarian tumors (14/14 (100%) versus 11/14 (78%)), lymph node metastasis (75/75 (100%) versus 60/75 (80%)), and peritoneal and pleural metastasis (9/9 (100%) versus 5/9 (56%). These findings suggest that ^68^Ga-FAPI PET/CT could supplement the diagnostic information provided by ^18^F-FDG PET/CT.

The peritoneum is the most common metastatic site and the main clinical challenge in ovarian cancer. Liu et al. (58) evaluated the diagnostic performance of the SUVmax and TBR of ^18^F-FDG and ^68^Ga-FAPI PET/CT in 29 patients with suspected platinum-sensitive recurrent ovarian cancer. Compared with the TBR of ^18^F-FDG, the TBR of ^68^Ga-FAPI PET/CT had superior sensitivity (95.83% versus 47.83%) and specificity (90.32% and 53.57%) for recurrent ovarian lesions and could therefore be used to assess the treatment response in platinum-sensitive recurrent ovarian cancer. The ^68^Ga-FAPI PET/CT displayed a huge advantage in peritoneal metastasis examination than ^18^F-FDG PET/CT. And in a study of 49 patients with epithelial ovarian cancer, Chen et al. (59) used surgical pathology to evaluate and compare the diagnostic performance of ^18^F-FDG and ^68^Ga-FAPI-04 PET/CT. They concluded that ^68^Ga-FAPI-04 PET/CT achieved higher sensitivity than ^18^F-FDG PET/CT in the detection and diagnosis of lymph node and peritoneal metastases, suggesting advantages regarding the preoperative staging of patients with EOC and, thereby, improving treatment decision-making.

### Applications of FAPI PET/CT in the treatment of ovarian cancer

Lymph node staging is a key step in diagnosing and treating patients with malignant ovarian tumors. Primary detumescence surgery and platinum-based adjuvant chemotherapy are the two main treatment options for ovarian cancer. However, about 80% of ovarian cancer patients relapse after treatment (60). Lindner T et al. (61) have developed a novel variant of FAPI by chelating it with technetium-99m (^99m^Tc-FAPI-34) for single photon emission computed tomography (SPECT) imaging of ovarian cancer. The resulting ^99m^Tc-labeled FAPI tracers revealed excellent binding properties (up to 45% binding, above 95% internalization), high affinity, and significant tumor uptake in biodistribution studies. This study also reported on a patient with metastasized ovarian cancer and a patient with pancreatic cancer that both received 6GBq ^90^Y-FAPI-46 as a last-line treatment. Significant tumor uptake was seen, with low uptake in healthy organs. The chelating agent used in this compound could also be labeled with the radionuclide rhenium-188(^188^Re). However, further research is required to evaluate this new radioisotope’s efficacy in treating ovarian cancer. In 2021, Kuyumcu et al. (62) published a dosimetry study where a low dose of ^177^Lu-FAPI-04 (267.5 ± 8.6 MBq) was given to four patients with metastatic advanced-stage cancer (breast cancer, thymic carcinoma, thyroid cancer, and ovarian cancer). Data acquisition was obtained using whole-body SPECT/CT imaging, and blood samples were collected for bone marrow dosimetry. The study shows that the estimated absorbed radiation dose to critical organs is significantly low with ^177^Lu-FAPI-04 compared with clinically established peptide-based radionuclide therapies. However, the authors concluded that employing different radioisotopes may be preferred due to the short tumor retention time of FAPI-04. Moreover, development of tracers with better tumor retention was warranted. Baum RP et al. (44) performed ^177^Lu-FAP-2286 for peptide-targeted radionuclide therapy (PTRT) in 11 patients with advanced adenocarcinomas of pancreas, breast, rectum, and ovary after prior confirmation of uptake on ^68^Ga-FAP-2286 or ^68^Ga-FAPI-04 PET/CT. Administration of ^177^Lu-FAP-2286 was well tolerated, with no adverse symptoms or clinically detectable pharmacologic effects being noticed or reported in any of the patients. It provided evidence of the feasibility of treating different aggressive adenocarcinomas with ^177^Lu-FAP-2286. These findings still strongly encourage additional research regarding FAPI and ovarian cancer, since ovarian cancer still lacks sufficient early diagnostic and therapeutic options.

## Cervical cancer

### Applications of FAPI PET/CT in the diagnosis of cervical cancer

Cervical cancer is the fourth most commonly diagnosed cancer and the fourth leading cause of cancer death in women all over the world. In 2020, there were an estimated 604,000 new cases of cervical cancer and 324,000 deaths worldwide, of which nearly 90% occurred in low- and middle-income countries (22, 63). The human papillomavirus (HPV) is one of the main causes of cervical cancer (22). From 2012 to 2019, the incidence of cervical cancer in women over 20 years old who received the HPV vaccine decreased by 65%, indicating a significant decline in the number of cervical cancer cases caused by HPV (63). However, 15–61% patients diagnosed with cervical cancer will subsequently go on to develop metastatic diseases (64). Therefore, early diagnosis and staging of cervical cancer are necessary. Squamous cell carcinoma is the most common histological cervical cancer. Other histological subtypes include adenocarcinoma, adenosquamous carcinoma, undifferentiated carcinoma, and neuroendocrine carcinoma. A biopsy is necessary to obtain a definitive diagnosis of cervical cancer. However, ^18^F-FDG PET/CT is often used to monitor disease progression and lymph node involvement after chemotherapy (65). Since cervical cancer often expresses FAP, radiolabeled FAPI is increasingly being used to diagnose and stage the disease. Wegen et al. (66) retrospectively reviewed 7 patients with histologically proven cervical cancer. All 7 patients had focal uptake above background in their tumor lesions in ^68^Ga-FAPI-46 PET/CT. The results showed that ^68^Ga-FAPI-46 PET/CT showed a higher TBR than ^18^F-FDG PET/CT in primary tumor as well as in lymph nodes metastasis. The higher TBR eventually improved the detection of both the primary tumor and lymph node metastasis in cervical cancer patients.

### Applications of FAPI PET/CT in the treatment of cervical cancer

Surgical resection and radiotherapy have an important role in the management of early-stage cervical cancer. However, patients with more advanced disease are treated with palliative chemotherapy. Although no studies were found evaluating the efficacy of radiolabeled FAPI in the treatment of cervical cancer, radioimmunotherapy drugs for HPV-positive cervical cancer show promising results in the management of cervical cancer. Phaeton R et al. (67) reported on the efficacy of using ^188^Re and ^177^Lu labeled monoclonal antibodies C1P5-E6 in CasKi cervical cancer xenograft nude mice. The findings of these studies have shown that these radioimmunotherapy drugs can inhibit tumor growth. However, clinical trials are required to confirm the efficacy of this treatment in improving tumor control and survival in patients diagnosed with advanced, recurrent, and metastatic cervical cancer.

## Endometrial cancer

### Applications of FAPI PET/CT in the diagnosis of endometrial cancer

Endometrial cancer is the sixth most common cancer in women worldwide. In 2020, the global incidence of endometrial cancer was 417,337 cases (22). Approximately 70% of patients with endometrial cancer are confined to the uterine body at the time of diagnosis, with early stage and good prognosis. Although the overall treatment effect of endometrial cancer is good, up to 15-20% of patients have relapsed (68). Endometrial cancer is categorized into type I and type II. Type I mostly consists of grade I or grade II endometrioid adenocarcinomas, which are sensitive to estrogen and often accompanied by endometrial hyperplasia in the early stages. Patients with this type cancer have favorable prognoses. Type II, on the other hand, comprises grade III endometrioid adenocarcinoma, clear serous carcinoma, undifferentiated carcinoma, and carcinosarcoma. These tumors are not influenced by estrogen or obesity and are associated with a worse prognosis (69). However, the pathological classification of endometrial cancer based on grading and histotype might varies for different observers (70, 71). ^18^F-FDG PET/CT is commonly used to evaluate the stage of endometrial carcinoma and has shown high performance in diagnosing preoperative lymph node metastasis and postoperative recurrence (72). However, the use of radiolabeled FAPI in the diagnosis and staging of endometrial cancer remains controversial. Studies have shown that the high uptake of the radiolabeled FAPI within normal uterine tissue and the low uptake in metastatic endometrial lesions limit its diagnostic efficacy (73). A high uptake of ^18^F-FDG within the normal uterus was reported in most women (66.7%) and was negatively correlated with age (18). Conversely, the uptake of ^68^Ga-FAPI-04 PET/CT was higher than that of ^18^F-FDG PET/CT in the metastatic lesions of some patients diagnosed with clear cell endometrial cancer (74). These findings suggest that FAPI PET/CT combined with ^18^F-FDG PET/CT may improve the diagnostic accuracy of primary and metastatic endometrial cancer.

### Applications of FAPI PET/CT in the treatment of endometrial cancer

The main treatment options for endometrial cancer include surgery with or without radiotherapy, brachytherapy and chemotherapy (75). The recent identification of the molecular endometrial cancer subtypes has led to the development of targeted therapies, particularly for early recurrent endometrial cancer subtypes (76). However, there are relatively few studies on the use of radiolabeled FAPI in the treatment of endometrial cancer, and further research is required to evaluate its role in the management of endometrial cancer.

## Discussion

This narrative review is focused on the role of radiolabeled FAPI in patients with breast cancer, ovarian cancer, cervical cancer and endometrial cancer, aiming to summarize the main evidence on those specific cancer. FAPI uptake in normal hormone-responsive organs determined significant differences in terms of pre- and postmenopausal status. Previously conducted studies indicated that differences in breast parenchyma are based on dynamic physiologic processes of the inner environment in response to endogenous and exogenous factors such as increased or decreased hormonal stimulations (77). The literature review demonstrates that ovary has no physiological FAPI uptake and there is no association with menstrual cycle. And changes in hormone response will influence FAPI uptake to a different extent in breast, endometrium, and uterus (11, 21). A series of histopathologic analyses demonstrated strong-to-moderate FAP expression is present in the stroma of breast carcinomas (78, 79). Differential uptake in hormone-sensitive organs may be a challenging fact as it may also be induced by physiological or benign conditions. Whether FAPI can overcome the assessment of potential cancers in hormone-sensitive organs, which is severely limited on FDG PET/CT, is unknown.

Besides, early response evaluation during and after therapy is possible with ^68^Ga-FAPI PET/CT scans, as FAP is part of the tumor microenvironment and molecular changes in the tumor stroma can subsequently be depicted (80). Consequently, these scans may allow precise monitoring, for example, after radiotherapy, which improves staging and further radiotherapy planning (81). As a visualization tool, FAPI has its own limitations compared to FDG, especially the accumulation observed in inflammatory and other non-tumorigenic conditions, which can further affect the diagnostic efficacy of FAPI. **Table 1** shows the comparison studies on the diagnostic performance of ^68^Ga-FAPI PET/CT and ^18^F-FDG PET/CT. For FAPI image interpretation, there are several pitfalls include non–tumor-specific ^68^Ga-FAPI uptake in the mammary glands, or the uterus. Pitfalls may originate from specific uptake through an increased FAP expression level and mechanisms of unspecific uptake, including edema, tracer extravasation, and some inflammatory disorders (18). Remarkably, FAPI, a single molecule, is both a diagnostic and possibly therapeutic agent, enabling additional theranostic application. However, literature evidence about the diagnosis of FAPI in breast cancer patients and the most common gynecologic malignancies has rapidly grown recently, to a major extent in the diagnostic use of ^68^Ga-labeled FAPI PET/CT at staging, restaging or differential diagnosis, but with few reports in the therapeutic perspective. **Table 2** shows the comparison studies on the theranostic performance of FAPI. Data was reported on breast cancer and the most common gynecologic malignancies patients that were treated with different FAP targeted radionuclide therapies shows that FAP targeted radionuclide therapy has resulted in objective responses in difficult to treat end stage cancer patients with manageable adverse events. Although no prospective data is yet available, these early data encourages further research (82). However, as a theranostic tool, FAPI’s relatively low blood retention necessitates modifications for enhancing therapeutic efficacy (14, 83). This is an important part of the issues that need to be addressed in future research on FAPI. More structural variants of FAPI will be needed in the future to ameliorate a number of problems with existing FAPI structures.

**Table 1:** Comparison studies on the diagnostic performance of ^68^Ga-FAPI PET/CT and ^18^F-FDG PET/CT.

First Author	Country	Journal	Patients (n)	Age (Years)	Year of Publication	Cancer Type	PET Image Analysis	^68^Ga-FAPI > ^18^F-FDG	^68^Ga-FAPI ≤ ^18^F-FDG
Zheng W (57)	China	Nucl Med Commun	27	18 or older	2023	Ovarian malignancies	/	-Lower background uptake and higher TBR (median TBR, 5.8 vs.2.7, P < 0.001) in primary ovarian tumors. -Higher SUVmax (median SUVmax, 7.0 vs. 4.4, P = 0.01) in lymph node metastases. -Higher SUVmax values in peritoneal and pleural metastases (median SUVmax, 10.1 vs. 7.6, P = 0.03), especially in pleural metastases.	-No evidence of a significant difference in bone and visceral metastases (median SUVmax, 6.9 vs.7.4, P = 0.14).
Liu S (58)	China	Eur J Nucl Med Mol Imaging	29	Age > 18 and < 80 years	2023	Platinum-sensitive recurrent ovarian	Semiquantitative image(^68^Ga-FAPI and ^18^F-FDG)	-Higher TBR(7.50 ± 6.42 vs. 3.19 ± 2.96, p < 0.001) in lesions uptake.-Higher sensitivity and accuracy(96.3% vs.49.07% and 97.40% vs. 63.87%, respectively) in lesion detection. -Higher TBR(11.13 ± 9.59 vs. 3.24 ± 3.12, p < 0.001) in lymph nodes detection. -A larger tumor burden (27 vs. 16, p = 0.025) according to the Eisenkop score.	-Similar SUVmax (4.49 ± 3.22 vs. 7.38 ± 6.39, p = 0.092) in lesions uptake. -The detection capability of lymph node metastasis was not clearly different.
Wegen S (66)	Germany	Clin Nucl Med	7	34-67	2023	Cervical Cancer	/	-Better TBRs in both primary tumor and nodal metastasis. -Better nodal tumor detection	/
Chen J (59)	China	Eur J Nucl Med Mol Imaging	49	51-66	2023	Epithelial ovarian cancer	Semi-quantitative(^68^Ga-FAPI and ^18^F-FDG)	-Outperform in detecting peritoneal and LN metastases. More sensitive in diagnosing peritoneal metastases(97.5% vs. 75.9%; p < 0.001) and LN metastases (80.6% vs. 61.3%; p = 0.031). -Higher uptake, higher volumetric parameters, and lower background activity in detecting peritoneal metastases. -Excellent diagnostic performance, with a sensitivity, specificity, and accuracy of 97.5%, 99.1%, and 98.5%, respectively.	-Both are comparable in the detection efficiency of primary ovarian tumors and distant metastases.
Kömek H (31)	Turkey	Ann Nucl Med	20	32-65	2021	Breast cancer	Semiquantitative (^68^Ga-FAPI and ^18^F-FDG)	-Higher sensitivity (100% ^18^F-FDGvs. 78.2% ^18^F-FDG), higher SUVmax, and higher TBR in detecting primary BC. -Lower background activity and higher uptake in subcentimetric lesions -Lower physiological uptake in liver, bone and brain	-Not a significant difference in SUVmax values of hepatic metastases (p > 0.05) -Similar specificity in detecting primary BC (100% ^18^F-FDG vs.95.6% ^68^Ga-FAPI)
Ballal S (16)	India	Eur J Nucl Med Mol Imaging	20	30-66	2021	Breast cancer and ovarian cancer	Qualitative and semiquantitative (^68^Ga-FAPI and ^18^F-FDG)	-A remarkably higher SULpeak and SULavg brain metastases-tobrain normal parenchyma ratios were observed on ^68^Ga-FAPI in contrast to the ^18^F-FDG. -An outstandingly high uptake was noted on ^68^Ga-FAPI PET/CT scans compared to the ^18^F-FDG PET/CT scans (P < 0.0001)	-Complete concordance in detecting primary residual tumor uptake and lymph node metastases. -Both the tracers identified all sites of skeletal metastases in all patients.
Elboga U (30)	Turkey	Ann Nucl Med	48	53.3 ± 11.7	2021	Breast cancer	Semiquantitative analysis (^68^Ga-FAPI and ^18^F-FDG)	-^68^Ga-FAPI was determined to be superior to ^18^F-FDG in terms of accuracy and sensitivity as well as high SUVmax values, and improved TBRs. -^68^Ga-FAPI detected lesions even within the first month of post-chemotherapy period than ^18^F-FDG	/
Dendl K (11)	Germany	Eur J Nucl Med Mol Imaging	Median 59.5	31	2021	Breast cancer, ovarian cancer,cervical cancer, and endometrial cancer	Semiquantitative (^68^Ga-FAPI and ^18^F-FDG)	-Mean SUVmax in all metastatic lesions of ^68^Ga-FAPI and ^18^F-FDG was slightly higher for FAPI accumulation than for FDG (8.2 vs 7.8; p = 0.131). -Slightly advantageous TBRs in regional lymph node metastases (31.9 vs 27.4; p = 0.6) and significantly in distant metastases (13.0 vs 5.7; p = 0.047). -FAPI accumulation in the ovaries presented no statistically significant differences pre- and postmenopausal (2.8 vs 1.6; p = 0.14)	-Higher ^18^F-FDG uptake in lung metastases (13.7 vs. 6.6; p=0.18)

**Table 2:** Comparison studies on the theranostic performance of FAPI.

First Author	Country	Journal	Year of Publication	Cancer Type	Therapeutic FAPI Tracer	Study Design	Patients	Treatment Response	Adverse Events
Baum RP (44)	Germany	J Nucl Med	2022	Breast cancer and ovarian cancer	^177^Lu-FAP-2286	Retrospective	5	-2 patients revealed stable disease -3 revealed progressive disease	-Short-term and self-limiting headache, moderate headache ,anemia and leukocytopenia
Assadi M (45)	Iran	Clin Nucl Med	2021	Breast cancer, cervical cancer and ovarian cancer	^177^Lu-FAPI-46	Prospective	8	-5 patients showed stable disease -3 showed progressive disease	-Increased bone pain
Ballal S (41)	India	Pharmaceuticals (Basel)	2021	Breast cancer	^177^Lu-DOTA.SA.FAPi [^177^Lu]Lu-DOTAGA.(SA.FAPi)2	Prospective	4	-All patients in two group have demonstrated a clinical response	-None of the patients experienced any early adverse events after the administration of the agents.
Rathke H (46)	Germany	Clin Nucl Med	2021	Breast cancer	^90^Y-FAPI-46	Prospective	1	-Stable disease	/
Lindner T (61)	Germany	J Nucl Med	2020	Ovarian cancer	^90^Y‐FAPI‐46	Prospective	2	-2 patients showed stable disease	/

## Conclusion

**Figure 1:**
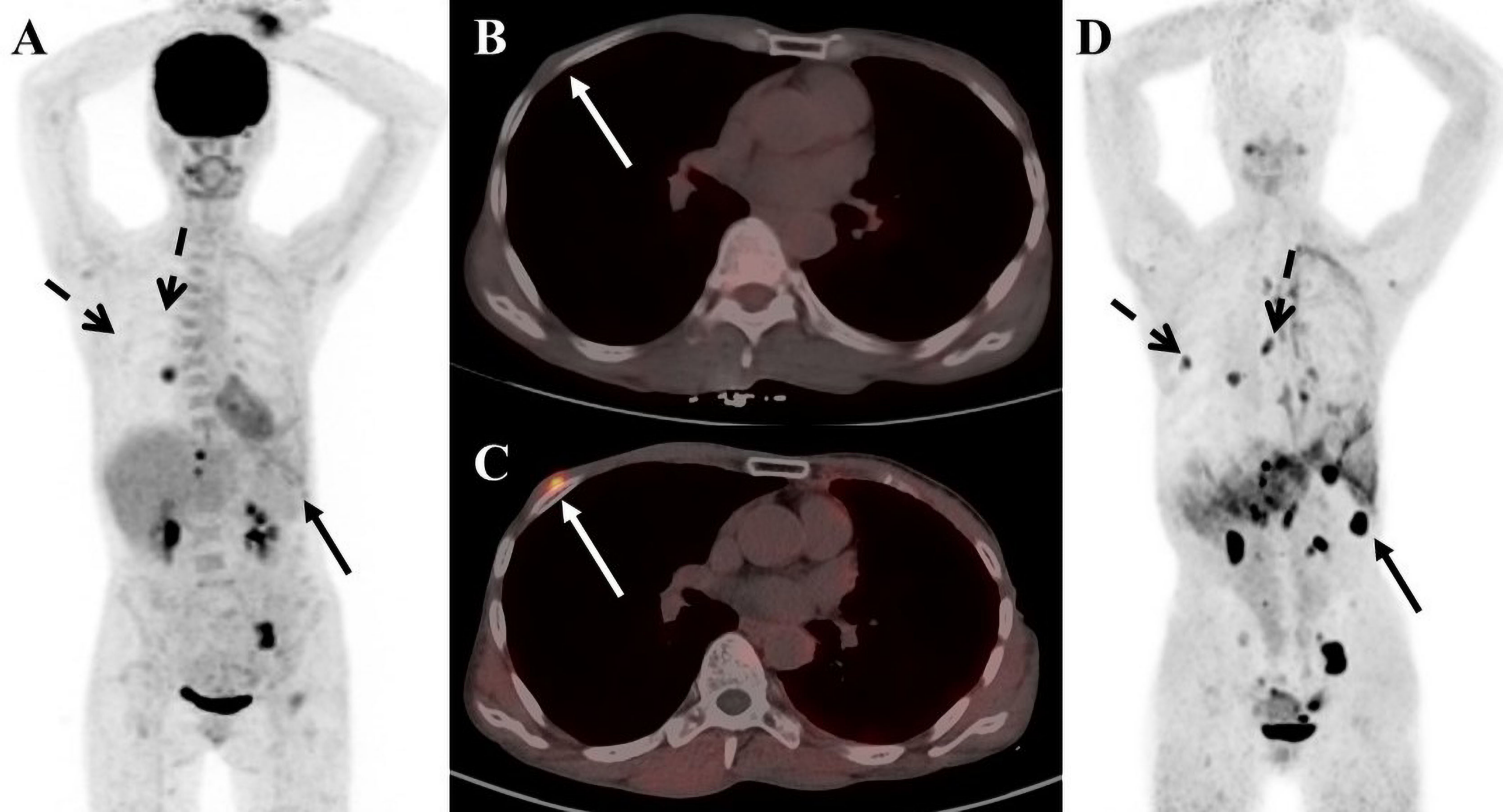
A 56-year-old female patient with breast cancer. **(A)** ^18^F-FDG PET/CT maximum-intensity-projection (MIP) image shows increased ^18^F-FDG uptake in the middle lobe of the right lung, the left ilium, the left pubic bone, and the left 11 ribs. **(B)** ^18^F-FDG PET/CT axial fusion chest image shows a slight thickening of the right chest wall, and no increase in ^18^F-FDG uptake. **(C)** FAPI PET/CT chest axial fusion image shows an increase in FAPI uptake in the right chest wall(SUVmax 7.3; white arrow). **(D)** FAPI PET/CT MIP image reveals a significant increase in imaging agent uptake in the in multiple lymph nodes, left lobe of liver and spleen parenchyma, without an increase in FDG uptake at the corresponding sites (black solid arrow and black dashed arrow).

**Figure 2:**
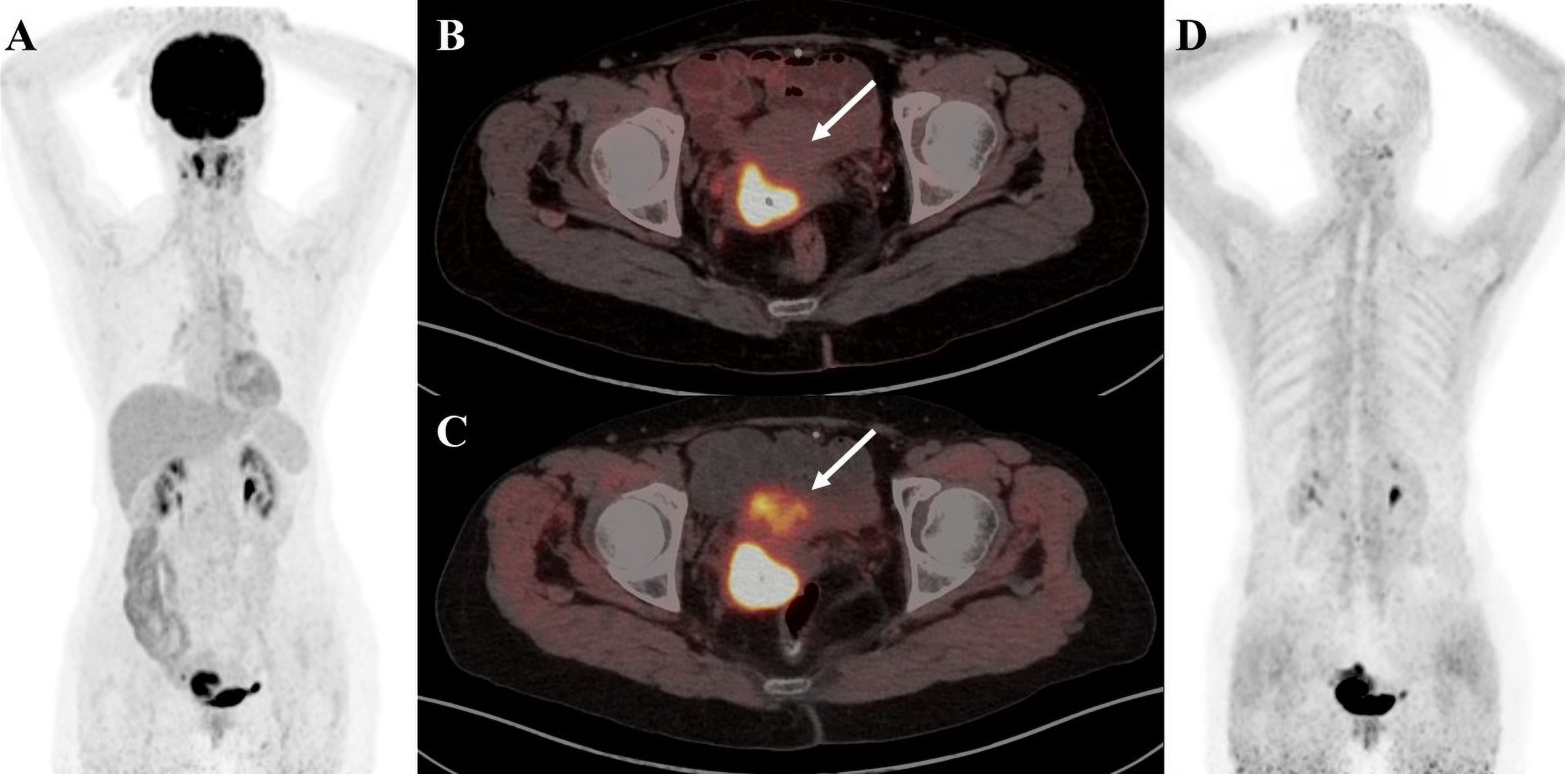
A 58-year-old female patient with cervical cancer was diagnosed as high-grade squamous intraepithelial lesion by cervical biopsy. **(A)** ^18^F-FDG PET/CT MIP image shows increased ^18^F-FDG uptake in the nasopharyngeal wall of the right pharyngeal recess and the bilateral palatine tonsils. **(B)** ^18^F-FDG PET/CT axial fusion image and **(C)** FAPI PET/CT axial fusion image show irregular thickening of the cervical wall, a mass-like shape, about 4.4cm × 2.3cm × 3.2cm in size, and increased uptake of imaging agent (SUVmax-FDG 14.3 vs SUVmax-FAPI 20.1, white arrow). **(D)** FAPI PET/CT MIP image reveals a significant increase in imaging agent uptake in the multiple lymph nodes, left lobe of liver and spleen parenchyma, without an increase in FDG uptake at the corresponding sites (black arrow).

**Figure 3:**
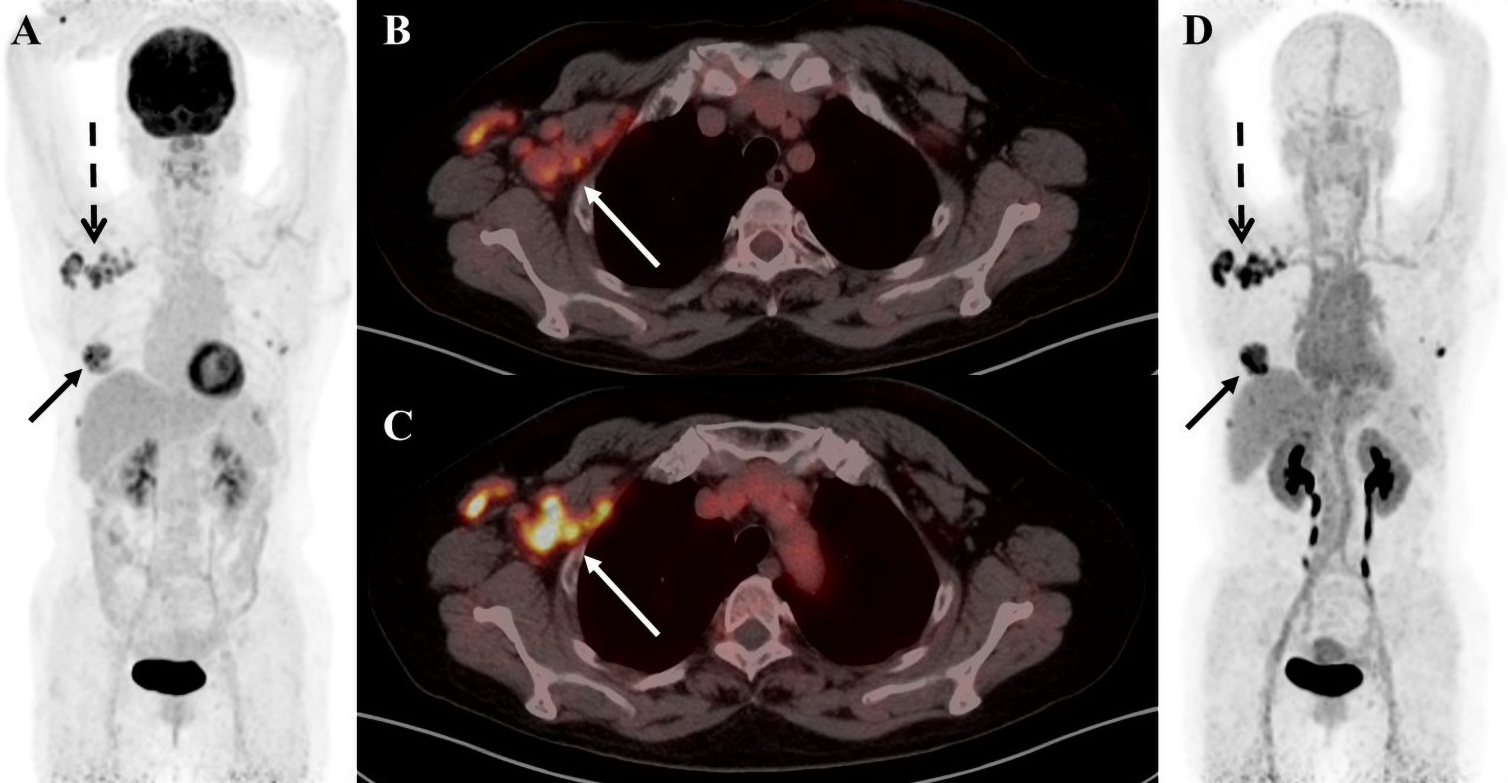
A 55-year-old female patient was diagnosed with breast carcinoma. **(A)** ^18^F-FDG PET/CT MIP image reveals a significant increase in imaging agent uptake in the right breast and multiple lymph nodes around the right axilla. **(B)** ^18^F-FDG PET/CT axial fusion image and **(C)** FAPI PET/CT axial fusion image show multiple enlarged lymph nodes around the right axilla and right pectoralis minor muscle, the larger measuring approximately 2.1 cm × 1.9 cm(SUVmax-FDG 4.5 vs SUVmax-FAPI 8.4, white arrow). **(D)** FAPI PET/CT MIP image reveals a significant FAPI uptake increase than FDG in multiple lymph nodes behind the right papilla, in the right axilla and around the right pectoralis minor(black solid arrow and black dashed arrow).

**Figure 4:**
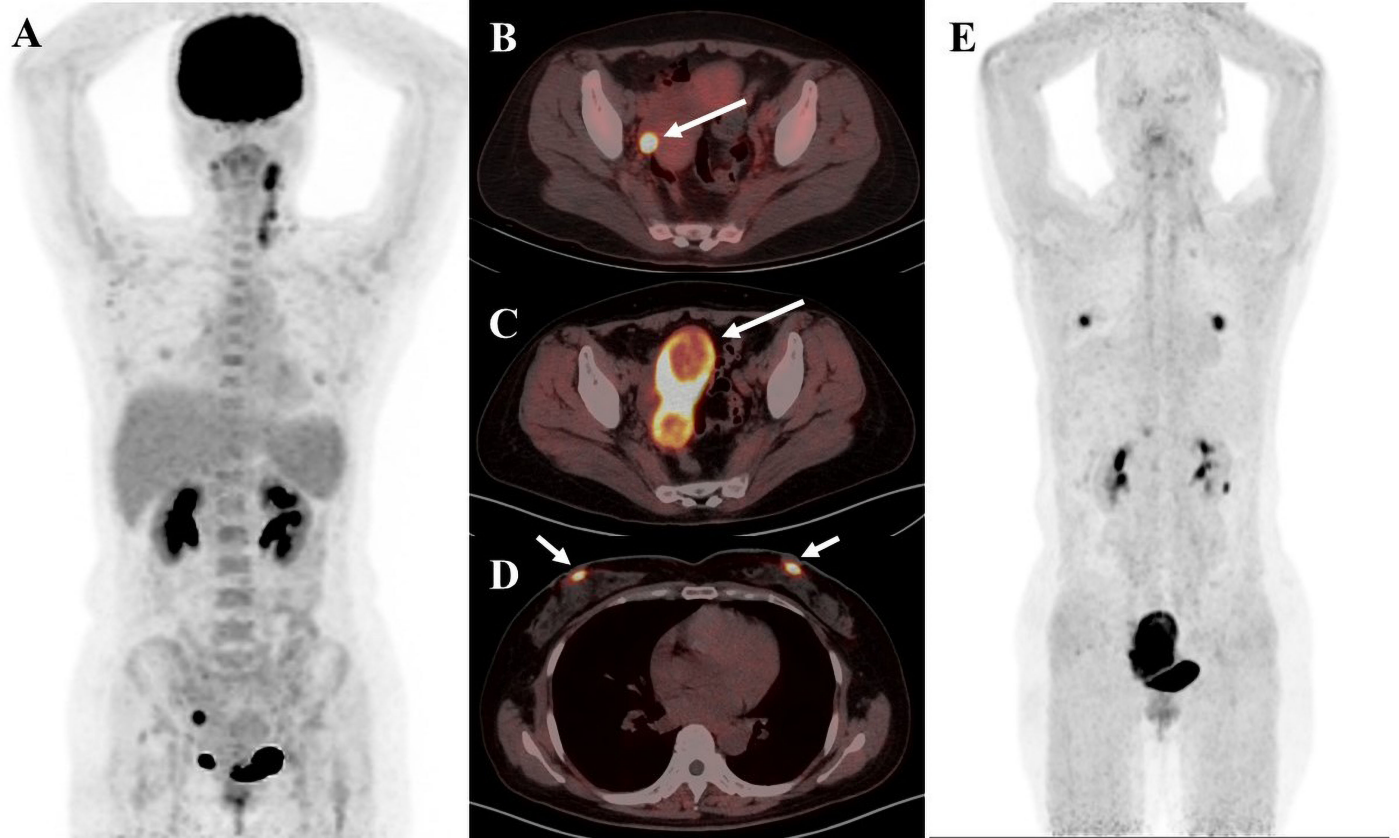
A 41-year-old female patient diagnosed with cervical cancer. **(A)** ^18^F-FDG PET/CT MIP image shows increased uptake of enlarged multiple lymph nodes in the left posterior cervical triangle and left supraclavicular region as well as nodular foci of increased glucose metabolism in the right adnexal region **(B)** ^18^F-FDG PET/CT axial fusion image and **(C)** FAPI PET/CT axial fusion image show nodular foci of increased contrast uptake are seen in the right adnexal region of the uterus(SUVmax-FDG 12.8 vs SUVmax-FAPI 14.5, white arrow). **(D)** FAPI PET/CT axial fusion image shows physiologic FAPI uptake exists in the mammary gland.

In this review, we discussed the applications of FAPI PET/CT in the diagnosis and treatment of breast and the most common gynecologic malignancies. Radiolabelled FAPI may improve the detection, staging, and assessment of treatment response in breast and the most common gynecologic malignancies, particularly when combined with ^18^F-FDG. In addition, it can also facilitate the differentiation between malignant and other benign lesions caused by inflammatory, infectious, and fibrotic diseases. For normal hormone-responsive organs, changes in hormone response will influence FAPI uptake to a different extent in breast, endometrium, and uterus. Therefore, hormonal effects are also an important factor that should be emphasized in FAPI applications. While the use of radiolabeled FAPI treatment for breast cancer has been extensively researched, investigations into its efficacy for the most common gynecologic malignancies remain in the preliminary stages. Therefore further research is required to confirm the clinical efficacy of this treatment (**Figures 1**–**4**).

## Author contributions

TL: Conceptualization, Visualization, Writing – original draft, Writing – review & editing. JZ: Visualization, Writing – review & editing. YY: Visualization, Writing – review & editing. MT: Visualization, Writing – review & editing. YC: Conceptualization, Writing – original draft, Writing – review & editing, Visualization.
